# Synthesis, crystal structure and Hirshfeld surface analysis of diethyl 2,6-dimethyl-4-(thio­phen-3-yl)-1,4-di­hydro­pyridine-3,5-di­carboxyl­ate

**DOI:** 10.1107/S2056989019015081

**Published:** 2019-11-15

**Authors:** Trung Vu Quoc, Duong Tran Thi Thuy, Thanh Phung Ngoc, Manh Vu Quoc, Hien Nguyen, Linh Duong Khanh, Anh Tu Quang, Luc Van Meervelt

**Affiliations:** a Faculty of Chemistry, Hanoi National University of Education, 136 Xuan Thuy, Cau Giay, Hanoi, Vietnam; b Bien Hoa Gifted High School, 86 Chu Van An Street, Phu Ly City, Ha Nam Province, Vietnam; c Phan Boi Chau Gifted High School, 119 Le Hong Phong Street, Vinh City, Nghe An Province, Vietnam; d Faculty of Foundation Science, College of Printing Industry, Phuc Dien, Bac Tu Liem, Hanoi, Vietnam; e Department of Chemistry, KU Leuven, Biomolecular Architecture, Celestijnenlaan 200F, Leuven (Heverlee), B-3001, Belgium

**Keywords:** crystal structure, hydrogen bonding, Hirshfeld analysis, 1,4-di­hydro­pyridine

## Abstract

In the title compound, C_17_H_21_NO_4_S, the 1,4-di­hydro­pyridine ring has an envelope conformation with the C*sp* ^3^ atom at the flap. The thio­phene ring is nearly perpendicular to the best plane through the 1,4-di­hydro­pyridine ring, the dihedral angle being 82.19 (13)°. In the crystal, chains running along the *b*-axis direction are formed through N—H⋯O inter­actions between the 1,4-di­hydro­pyridine N atom and one of the O atoms of the ester groups. Neighbouring chains are linked by C—H⋯O and C—H⋯π inter­actions. A Hirshfeld surface analysis shows that the most prominent contributuion to the surface contacts are H⋯H contacts (55.1%).

## Abstract 2

In the title 1,4-di­hydro­pyridine derivative, the 1,4-di­hydro­pyridine ring makes an angle of 82.19 (13)° with the thio­phene ring. In the crystal, N—H⋯O and C—H⋯O hydrogen bonds as well as C—H⋯π inter­actions link the mol­ecules into a three-dimensional network.

## Chemical context   

1,4-Di­hydro­pyridine derivatives exhibit a large range of biological activities (Stout & Meyers, 1982 ▸; Wei *et al.*, 1989 ▸; Bossert & Vater, 1989 ▸; Mauzerall & Westheimer, 1955 ▸). They have been used as anti­convulsant, anti­depressive, anti­anxiety, analgesic, anti­tumoral, vasodilator and anti-inflammatory agents (Sausins & Duburs, 1988 ▸; Boecker & Guenguerich, 1986 ▸; Godfraind *et al.*, 1986 ▸). Some of them, such as amlodipine, felodipine and isradipine are drugs effective as calcium-channel blockers for the treatment of cardiovascular diseases and hypertension (Bossert *et al.*, 1981 ▸; Nakayama & Kanoaka, 1996 ▸; Gordeev *et al.*, 1996 ▸). 1,4-Di­hydro­pyridines are also good precursors of the corresponding substituted pyridine derivatives and constitute useful reducing agents for imines in the presence of a catalytic amount of Lewis acid (Xia & Wang, 2005 ▸; Heravi *et al.*, 2005 ▸; Bagley & Lubinu, 2006 ▸).
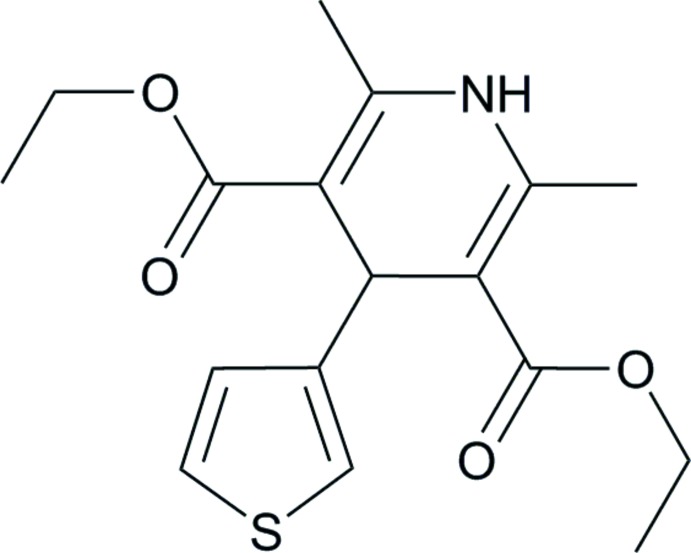


As a continuation of our research on the chemical and physical properties of novel polythio­phenes (Nguyen *et al.*, 2016 ▸; Vu *et al.*, 2016 ▸), some new thio­phene monomers have been prepared (Vu *et al.*, 2017 ▸, 2018 ▸, 2019 ▸; Nguyen *et al.*, 2017 ▸). In this study, the synthesis and crystal structure of diethyl 2,6-dimethyl-4-(thio­phen-3-yl)-1,4-di­hydro­pyridine-3,5-di­carb­ox­y­l­ate are presented together with a Hirshfeld surface analysis and non-covalent inter­action plots.

## Structural commentary   

**Figure 1:**
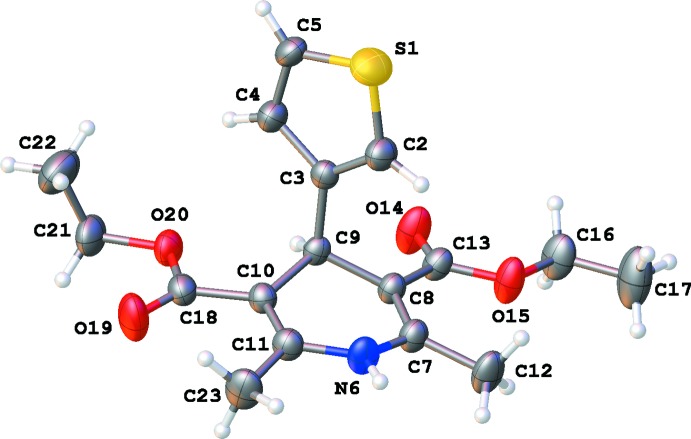
A view of the mol­ecular structure of the title compound, with atom labels and displacement ellipsoids drawn at the 50% probability level. H atoms are shown as small circles of arbitrary radii.

The title compound crystallizes in the monoclinic space group *P*2_1_/*c* with one mol­ecule in the asymmetric unit (Fig. 1 ▸). The 1,4-di­hydro­pyridine ring (N6,C7–C11) has an envelope conformation with atom C9 at the flap [puckering parameters: *Q* = 0.300 (3) Å, θ = 73.9 (6)°, φ = 182.0 (5)°]. The best plane through the 1,4-di­hydro­pyridine ring makes an angle of 82.19 (13)° with the plane through the thio­phene ring (S1/C2–C5; r.m.s. deviation = 0.001 Å). Both methyl C atoms are closer to the best plane through the 1,4-di­hydro­pyridine [deviations: C12 − 0.164 (3) Å, C23 − 0.162 (3) Å] than the C atoms of the two ester substituents [deviations: C13 − 0.363 (3) Å, C18 − 0.446 (2) Å]. All four of these C atoms are at the opposite sides with respect to the thio­phene substituent which is in an axial position. Atoms O15, O19 and O20 are involved in intra­molecular short contacts (Table 1 ▸). Both ester groups have a different conformation as illustrated by torsion angles C13—O15—C16—C17 [177.2 (3)°, +ap] and C18—O20—C21—C22 [85.3 (8)°, +sc].

## Supra­molecular features and Hirshfeld surface analysis   

**Figure 2:**
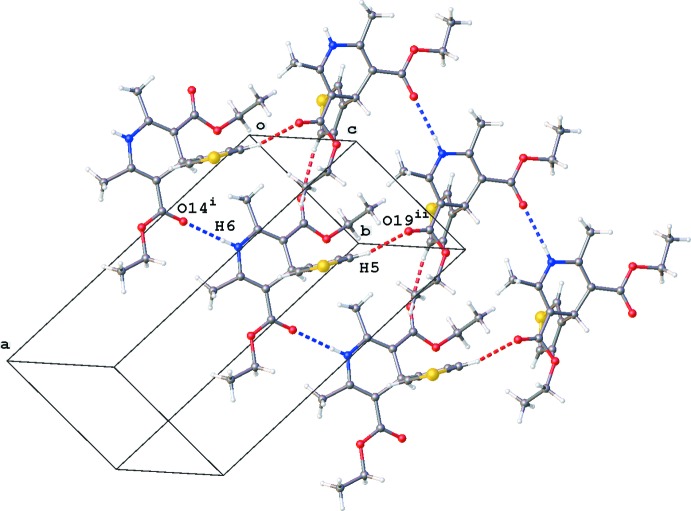
Partial crystal packing of the title compound, showing the chain formation along the *b* axis by N—H⋯O inter­actions (blue dashed lines). Parallel chains are linked by C—H⋯O inter­actions (red dashed lines; see Table 1 ▸ for symmetry codes).

**Figure 3:**
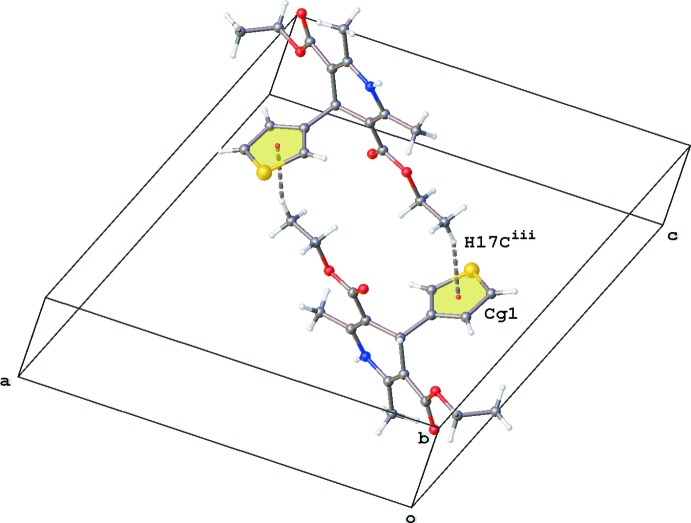
Partial crystal packing of the title compound, showing the inversion dimer formation through C—H⋯π inter­actions (grey dashed lines; *Cg*1 is the centroid of the S1/C2–C5 ring; see Table 1 ▸ for symmetry code).

In the crystal, the 1,4-di­hydro­pyridine N6 atom acts as a hydrogen-bond donor to the O14 atom of one of the ester groups, resulting in chain formation along the *b*-axis direction (Fig. 2 ▸, Table 1 ▸). Parallel chains are linked by C—H⋯O hydrogen bonds between the thio­phene H5 atom and the carbonyl O19 atom of the second ester group (Fig. 2 ▸, Table 1 ▸). In addition, inversion dimers are formed by C—H⋯π inter­actions (Fig. 3 ▸, Table 1 ▸). No voids are observed in the crystal packing of the title compound.

**Figure 4:**
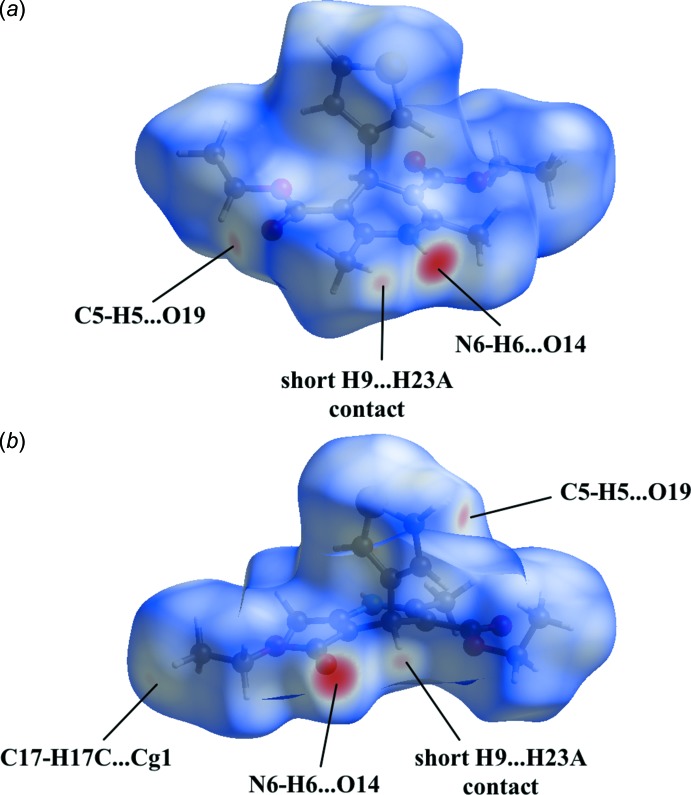
Two views of the Hirshfeld surface mapped over *d*_norm_ for the title compound in the range −0.4662 to +1.2830 arbitrary units.

**Figure 5:**
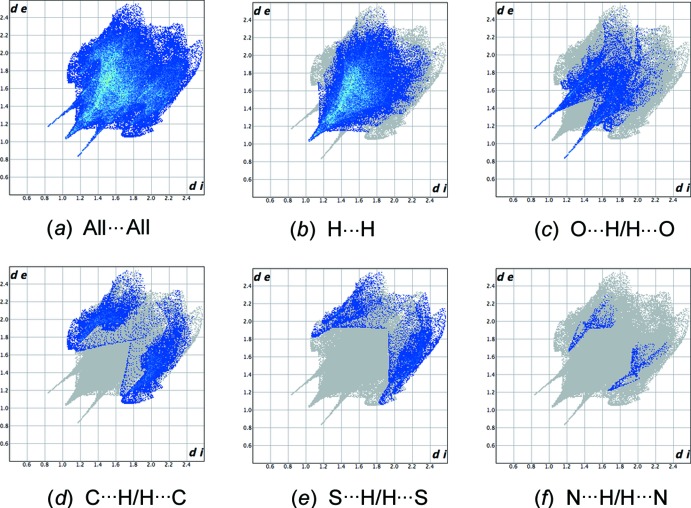
Full two-dimensional fingerprint plots for the title compound, showing (*a*) all inter­actions, and delineated into (*b*) H⋯H, (*c*) O⋯H/H⋯O, (*d*) C⋯H/H⋯C, (*e*) S⋯H/H⋯S, (*f*) N⋯H/H⋯N inter­actions. The *d*_i_ and *d*_e_ values are the closest inter­nal and external distances (in Å) from a given point on the Hirshfeld surface.

In order to gain further insight into the packing, the Hirshfeld surface and fingerprint plots were calculated using *CrystalExplorer* (Turner *et al.*, 2017 ▸). The Hirshfeld surface (Spackman & Jayatilaka, 2009 ▸) mapped over *d*_norm_ in Fig. 4 ▸ shows bright-red spots near the atoms participating in the already discussed inter­molecular inter­actions. In addition a faint-red spot is present near atoms H9 and H23*A* indicating a short H9⋯H23*A* ^iv^ contact distance of 2.276 Å [symmetry code: (iv) *x*, *y* + 1, *z*]. The associated two-dimensional fingerprint plots (McKinnon *et al.*, 2007 ▸) are shown in Fig. 5 ▸ and give additional information about the inter­molecular contacts. H⋯H Van der Waals contacts dominate (55.1%) and appear in the middle of the scattered points in the fingerprint plot (Fig. 5 ▸ *b*). The contribution (16.4%) from the O⋯H/H⋯O contacts shows a pair of sharp spikes corresponding to the N—H⋯O inter­actions (Fig. 5 ▸ *c*). In addition, C⋯H/H⋯C and S⋯H/H⋯S contacts contribute 15.7 and 9.6%, respectively, to the Hirshfeld surface. A further small contribution is from N⋯H/H⋯N contacts (1.5%, Fig. 5 ▸ *f*). The percentage contributions of the other contact types are negligible.

Enrichment ratios (Table 2 ▸) were calculated according to the method described by Jelsch *et al.* (2014 ▸). A ratio *E_XY_* greater than unity for a pair of elements *X* and *Y* indicates a high likelihood of forming *X*⋯*Y* contacts in the crystal packing. The favourable O⋯H and H⋯π contacts in the crystal packing are reflected in the enrichment ratios *E*_OH_ of 1.24 and *E*_CH_ of 1.23 for these contacts. The slight *E*_SH_ enrichment (1.11) refers to the multiple S⋯H contacts between S1 and neighbouring methyl groups (S⋯H distances ranging from 3.01 to 3.50 Å). However, the high enrichment ratio *E*_NH_ must be inter­preted with caution as it results from the quotient of two small numbers (Jelsch *et al.*, 2014 ▸).

## Database survey   

**Figure 6:**
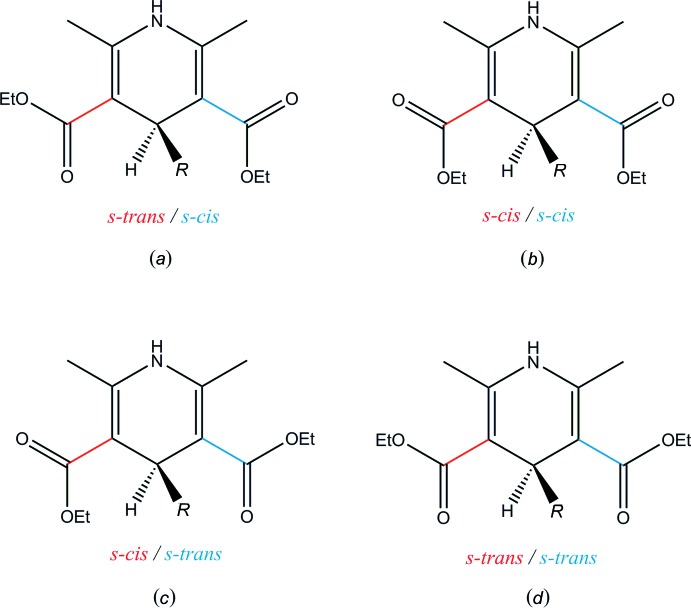
Four possible orientations of the C=O groups for diethyl 2,6-dimethyl-1,4-di­hydro­pyridine-3,5-di­carboxyl­ate derivatives with a ring substituent (*R*) at C4.

A search of the Cambridge Structural Database (CSD, Version 5.40, update of May 2019; Groom *et al.*, 2016 ▸) for diethyl 2,6-dimethyl-1,4-di­hydro­pyridine-3,5-di­carboxyl­ate derivatives with a ring substituent at C4 results in 70 hits for which coordinates are available. Most similar to the title compound is the 4-(2-thien­yl) derivative (refcode QIWWEY; Caignan *et al.*, 2000 ▸; refcode QIWWEY01; Huang & Cui, 2016 ▸). In these compounds the thienyl group is disordered over two sets of sites with an occupancy ratio of 0.51:0.49. An overlay between the title compound and QIWWEY excluding the thio­phene ring gives an r.m.s. deviation of 0.318 Å. In QIWWEY, the 1,4-di­hydro­pyridine and thio­phene rings make an angle of 83.19 (17)°. Fig. 6 ▸ shows the four possible orientations of the two C=O substituents on the 1,4-di­hydro­pyridine ring. Most popular are the *s-trans*/*s-cis* (35%), the *s-cis*/*s-cis* (31%) and the *s-cis*/*s-trans* conformation (29%). The *s-trans*/*s-trans* conformation occurs only for 5% of the deriv­atives. In the title compound, both C=O substituents are present in an *s-trans*/*s-cis* conformation.

## Synthesis and crystallization   

**Figure 7:**
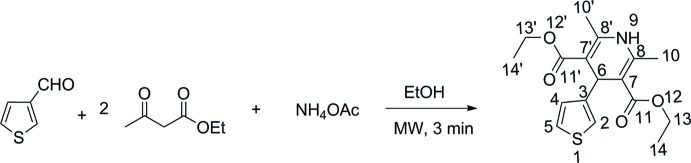
Reaction scheme for the synthesis of the title compound.

The reaction scheme for the synthesis of the title compound is given in Fig. 7 ▸.

***Synthesis of diethyl 2,6-dimethyl-4-(thio­phen-3-yl)-1,4-di­hydro­pyridine-3,5-di­carboxyl­ate*** **:**

A mixture of thio­phene-3-carbaldehyde (3 mmol), ethyl aceto­acetate (6 mmol) and NH_4_OAc (3 mmol) in ethanol (10 mL) was exposed to microwave radiation for 3 min. at a power of 450W. The reaction mixture was cooled down and the solid product was separated by filtration and purified by recrystallization in ethanol to give the compound as yellowish transparent crystals (yield 82%), m.p. 423 K. IR (KBr, cm^−1^): 3346, 3244 (NH), 3099, 2979 (C–H), 1699 (C=O), 1490 (C=C). ^1^H NMR [Bruker XL-500, 500 MHz, *d*_6_-CDCl_3_, δ (ppm), *J* (Hz), see Fig. 7 ▸ for numbering scheme]: 7.12 (*m*, 1H, *J =* 4.5, H^4^), 6.99 (*m*, 1H, *J* = 4.5, H^5^), 6.91 (*d*, 1H,, *J* = 2.5, H^2^), 5.93 (*s*, 1H, H^9^), 5.14 (*s*, 1H, H^6^), 4.13 (*m*, 4H, *J* = 7.5Hz, H^13,13′^), 2.30 (*s*, 6H, H^10,10′^), 1.25 (*m*, 6H, *J* = 7.25 H^14,14′^). ^13^C NMR [Bruker XL-500, 125 MHz, *d*_6_-CDCl_3_, (ppm)]: 19.4 (C^10,10′^), 14.3 (C^14,14′^), 34.6 (C^6^), 59.7 (C^13,13′^), 103.4 (C^7,7′^), 120.3 (C^2^), 124.6 (C^4^), 127.6 (C^5^), 144.4 (C^3^), 147.9 (C^8^,C^8′^), 167.7 (C^11,11′^). Calculated for C_17_H_21_NO_4_S: *M* ^[+H]^ = 335.4 au.

## Refinement   

Crystal data, data collection and structure refinement details are summarized in Table 3 ▸. H atom H6 was found in a difference electron-density map and refined freely. The other H atoms were placed in idealized positions and included as riding contributions with *U*_iso_(H) values of 1.2*U*_eq_ or 1.5*U*_eq_ of the parent atoms, with C—H distances of 0.93 (aromatic), 0.98 (CH), 0.97 (CH_2_) and 0.96 Å (CH_3_). In the final cycles of refinement, four outlying reflections were omitted.

## Supplementary Material

Crystal structure: contains datablock(s) I, global. DOI: 10.1107/S2056989019015081/sj5585sup1.cif

Structure factors: contains datablock(s) I. DOI: 10.1107/S2056989019015081/sj5585Isup2.hkl

Supporting information file. DOI: 10.1107/S2056989019015081/sj5585Isup3.cml

CCDC references: 1964486, 1964486

Additional supporting information:  crystallographic information; 3D view; checkCIF report

## Figures and Tables

**Table 1:** Hydrogen-bond geometry (Å, °) *Cg*1 is the centroid of the thio­phene S1/C2–C5 ring.

*D*—H⋯*A*	*D*—H	H⋯*A*	*D*⋯*A*	*D*—H⋯*A*
N6—H6⋯O14^i^	0.82 (4)	2.19 (4)	3.010 (3)	176 (3)
C5—H5⋯O19^ii^	0.93	2.52	3.220 (4)	133
C9—H9⋯O20	0.98	2.36	2.739 (3)	102
C12—H12*B*⋯O15	0.96	2.33	2.768 (3)	107
C23—H23*C*⋯O19	0.96	2.42	2.826 (4)	105
C17—H17*C*⋯*Cg*1^iii^	0.96	2.79	3.720 (4)	162

Symmetry codes: (i) 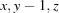; (ii) 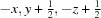; (iii) 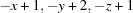.

**Table 2:** Enrichment ratios for the title compound

Parameter	Ratio
H⋯H	0.94
C⋯H	1.23
O⋯H	1.24
N⋯H	1.30
S⋯H	1.11
S⋯C	0.96
S⋯O	0.82

**Table 3:** Experimental details

Crystal data	
Chemical formula	C_17_H_21_NO_4_S
*M*_r_	335.41
Crystal system, space group	Monoclinic, *P*2_1_/*c*
Temperature (K)	293
*a*, *b*, *c* (Å)	15.6801 (8), 7.4311 (3), 15.5968 (8)
β (°)	111.424 (6)
*V* (Å^3^)	1691.77 (16)
*Z*	4
Radiation type	Mo *K*α
μ (mm^−1^)	0.21
Crystal size (mm)	0.5 × 0.2 × 0.05

Data collection	
Diffractometer	Rigaku Oxford Diffraction SuperNova, single source at offset/far, Eos
Absorption correction	Multi-scan (*CrysAlis PRO*; Rigaku OD, 2018 ▸)
*T*_min_, *T*_max_	0.555, 1.000
No. of measured, independent and observed [*I* > 2σ(*I*)] reflections	19775, 3446, 2805
*R*_int_	0.027
(sin θ/λ)_max_ (Å^−1^)	0.625

Refinement	
*R*[*F* ^2^ > 2σ(*F* ^2^)], *wR*(*F* ^2^), *S*	0.061, 0.187, 1.05
No. of reflections	3446
No. of parameters	216
H-atom treatment	H atoms treated by a mixture of independent and constrained refinement
Δρ_max_, Δρ_min_ (e Å^−3^)	0.53, −0.47

Computer programs: *CrysAlis PRO* (Rigaku OD, 2018 ▸), *SHELXT2014* (Sheldrick, 2015*a* ▸), *SHELXL2016* (Sheldrick, 2015*b* ▸) and *OLEX2* (Dolomanov *et al.*, 2009 ▸).

## supplementary crystallographic information

### Crystal data

**Table:**

C_17_H_21_NO_4_S	*F*(000) = 712
*M**_r_* = 335.41	*D*_x_ = 1.317 Mg m^−^^3^
Monoclinic, *P*2_1_/*c*	Mo *K*α radiation, λ = 0.71073 Å
*a* = 15.6801 (8) Å	Cell parameters from 8195 reflections
*b* = 7.4311 (3) Å	θ = 3.1–27.9°
*c* = 15.5968 (8) Å	µ = 0.21 mm^−^^1^
β = 111.424 (6)°	*T* = 293 K
*V* = 1691.77 (16) Å^3^	Block, colourless
*Z* = 4	0.5 × 0.2 × 0.05 mm

### Data collection

**Table:**

Rigaku Oxford Diffraction SuperNova, single source at offset/far, Eos diffractometer	3446 independent reflections
Radiation source: micro-focus sealed X-ray tube, SuperNova (Mo) X-ray Source	2805 reflections with *I* > 2σ(*I*)
Mirror monochromator	*R*_int_ = 0.027
Detector resolution: 15.9631 pixels mm^-1^	θ_max_ = 26.4°, θ_min_ = 2.6°
ω scans	*h* = −19→19
Absorption correction: multi-scan (CrysAlis PRO; Rigaku OD, 2018)	*k* = −9→9
*T*_min_ = 0.555, *T*_max_ = 1.000	*l* = −19→19
19775 measured reflections	

### Refinement

**Table:**

Refinement on *F*^2^	0 restraints
Least-squares matrix: full	Hydrogen site location: mixed
*R*[*F*^2^ > 2σ(*F*^2^)] = 0.061	H atoms treated by a mixture of independent and constrained refinement
*wR*(*F*^2^) = 0.187	*w* = 1/[σ^2^(*F*_o_^2^) + (0.0981*P*)^2^ + 1.416*P*] where *P* = (*F*_o_^2^ + 2*F*_c_^2^)/3
*S* = 1.05	(Δ/σ)_max_ < 0.001
3446 reflections	Δρ_max_ = 0.53 e Å^−^^3^
216 parameters	Δρ_min_ = −0.46 e Å^−^^3^

### Special details

**Table:**

Geometry. All esds (except the esd in the dihedral angle between two l.s. planes) are estimated using the full covariance matrix. The cell esds are taken into account individually in the estimation of esds in distances, angles and torsion angles; correlations between esds in cell parameters are only used when they are defined by crystal symmetry. An approximate (isotropic) treatment of cell esds is used for estimating esds involving l.s. planes.

### Fractional atomic coordinates and isotropic or equivalent isotropic displacement parameters (Å^2^)

**Table:**

	*x*	*y*	*z*	*U*_iso_*/*U*_eq_	
S1	0.23846 (6)	0.68160 (12)	0.53767 (5)	0.0587 (3)	
C2	0.28608 (18)	0.6398 (4)	0.45764 (17)	0.0407 (6)	
H2	0.340156	0.575481	0.470126	0.049*	
C3	0.23681 (15)	0.7104 (3)	0.37324 (15)	0.0293 (5)	
C4	0.15812 (17)	0.8010 (4)	0.37597 (18)	0.0405 (6)	
H4	0.115977	0.858331	0.325101	0.049*	
C5	0.14966 (17)	0.7959 (4)	0.46445 (18)	0.0393 (6)	
H5	0.102525	0.847582	0.478796	0.047*	
N6	0.32530 (15)	0.3391 (3)	0.28906 (16)	0.0406 (5)	
H6	0.346 (2)	0.236 (5)	0.297 (2)	0.053 (9)*	
C7	0.39001 (16)	0.4737 (3)	0.31863 (17)	0.0351 (5)	
C8	0.36158 (15)	0.6465 (3)	0.31363 (16)	0.0302 (5)	
C9	0.26034 (14)	0.6872 (3)	0.28729 (15)	0.0273 (5)	
H9	0.246735	0.800269	0.252562	0.033*	
C10	0.20195 (15)	0.5392 (3)	0.22653 (15)	0.0303 (5)	
C11	0.23455 (16)	0.3689 (3)	0.23509 (17)	0.0359 (5)	
C12	0.48666 (18)	0.4023 (4)	0.3531 (2)	0.0533 (8)	
H12A	0.509780	0.397134	0.419186	0.080*	
H12B	0.524659	0.480463	0.333367	0.080*	
H12C	0.487105	0.283768	0.328792	0.080*	
C13	0.42150 (16)	0.8049 (3)	0.33895 (17)	0.0348 (5)	
O14	0.39447 (13)	0.9578 (2)	0.32155 (16)	0.0545 (6)	
O15	0.50981 (12)	0.7683 (2)	0.38631 (16)	0.0516 (5)	
C16	0.57344 (18)	0.9188 (4)	0.4152 (2)	0.0532 (7)	
H16A	0.553963	1.002135	0.452336	0.064*	
H16B	0.575719	0.982674	0.361883	0.064*	
C17	0.6646 (2)	0.8441 (5)	0.4697 (3)	0.0831 (13)	
H17A	0.683923	0.764533	0.431803	0.125*	
H17B	0.661164	0.778721	0.521443	0.125*	
H17C	0.707965	0.940521	0.491285	0.125*	
C18	0.10811 (16)	0.5808 (4)	0.16274 (16)	0.0363 (5)	
O19	0.05131 (14)	0.4733 (3)	0.11903 (16)	0.0646 (7)	
O20	0.09182 (12)	0.7592 (2)	0.15614 (12)	0.0410 (4)	
C21	0.00100 (19)	0.8172 (5)	0.0952 (2)	0.0525 (7)	
H21A	−0.020354	0.738253	0.042024	0.063*	
H21B	0.005081	0.938003	0.073416	0.063*	
C22	−0.0661 (3)	0.8156 (6)	0.1416 (3)	0.0767 (11)	
H22A	−0.124239	0.858116	0.099750	0.115*	
H22B	−0.044950	0.892617	0.194641	0.115*	
H22C	−0.072715	0.695134	0.160476	0.115*	
C23	0.1849 (2)	0.2020 (4)	0.1900 (2)	0.0542 (8)	
H23A	0.218986	0.098199	0.220459	0.081*	
H23B	0.178354	0.200892	0.126401	0.081*	
H23C	0.125325	0.199968	0.194332	0.081*	

### Atomic displacement parameters (Å^2^)

**Table:**

	*U*^11^	*U*^22^	*U*^33^	*U*^12^	*U*^13^	*U*^23^
S1	0.0677 (5)	0.0662 (6)	0.0442 (4)	0.0079 (4)	0.0228 (4)	0.0059 (3)
C2	0.0385 (13)	0.0430 (14)	0.0386 (13)	0.0084 (11)	0.0117 (10)	0.0040 (11)
C3	0.0288 (10)	0.0250 (11)	0.0327 (11)	−0.0019 (8)	0.0094 (9)	−0.0028 (8)
C4	0.0339 (12)	0.0462 (14)	0.0391 (13)	0.0080 (11)	0.0104 (10)	−0.0014 (11)
C5	0.0313 (12)	0.0424 (14)	0.0466 (14)	0.0033 (10)	0.0169 (11)	−0.0069 (11)
N6	0.0372 (11)	0.0225 (10)	0.0547 (13)	0.0035 (8)	0.0082 (10)	0.0007 (9)
C7	0.0298 (11)	0.0306 (12)	0.0422 (13)	0.0026 (9)	0.0101 (10)	0.0014 (10)
C8	0.0266 (11)	0.0267 (11)	0.0362 (11)	0.0012 (8)	0.0100 (9)	0.0014 (9)
C9	0.0269 (10)	0.0209 (10)	0.0333 (11)	0.0021 (8)	0.0098 (9)	0.0024 (8)
C10	0.0289 (11)	0.0289 (11)	0.0314 (11)	−0.0019 (9)	0.0091 (9)	0.0004 (9)
C11	0.0350 (12)	0.0312 (12)	0.0399 (12)	−0.0025 (10)	0.0116 (10)	−0.0027 (10)
C12	0.0359 (14)	0.0342 (14)	0.080 (2)	0.0093 (11)	0.0095 (13)	−0.0009 (14)
C13	0.0295 (11)	0.0292 (12)	0.0465 (13)	0.0007 (9)	0.0146 (10)	0.0007 (10)
O14	0.0375 (10)	0.0254 (9)	0.0918 (15)	0.0001 (7)	0.0134 (10)	0.0048 (9)
O15	0.0300 (9)	0.0301 (9)	0.0830 (15)	−0.0027 (7)	0.0068 (9)	0.0011 (9)
C16	0.0378 (14)	0.0339 (14)	0.081 (2)	−0.0091 (11)	0.0137 (13)	0.0000 (14)
C17	0.0435 (17)	0.056 (2)	0.124 (3)	−0.0095 (15)	0.0001 (19)	0.009 (2)
C18	0.0321 (11)	0.0432 (14)	0.0312 (11)	0.0001 (10)	0.0084 (9)	−0.0009 (10)
O19	0.0429 (11)	0.0552 (13)	0.0715 (14)	−0.0041 (10)	−0.0079 (10)	−0.0129 (11)
O20	0.0338 (9)	0.0420 (10)	0.0395 (9)	0.0080 (7)	0.0043 (7)	0.0042 (7)
C21	0.0391 (14)	0.0634 (19)	0.0449 (15)	0.0146 (13)	0.0035 (12)	0.0102 (13)
C22	0.055 (2)	0.091 (3)	0.084 (3)	0.0277 (19)	0.0258 (18)	0.005 (2)
C23	0.0489 (16)	0.0326 (14)	0.073 (2)	−0.0062 (12)	0.0128 (14)	−0.0139 (13)

### Geometric parameters (Å, º)

**Table:**

S1—C2	1.701 (3)	C12—H12C	0.9600
S1—C5	1.674 (3)	C13—O14	1.208 (3)
C2—H2	0.9300	C13—O15	1.338 (3)
C2—C3	1.364 (3)	O15—C16	1.456 (3)
C3—C4	1.420 (3)	C16—H16A	0.9700
C3—C9	1.525 (3)	C16—H16B	0.9700
C4—H4	0.9300	C16—C17	1.479 (4)
C4—C5	1.434 (4)	C17—H17A	0.9600
C5—H5	0.9300	C17—H17B	0.9600
N6—H6	0.82 (4)	C17—H17C	0.9600
N6—C7	1.379 (3)	C18—O19	1.205 (3)
N6—C11	1.381 (3)	C18—O20	1.347 (3)
C7—C8	1.352 (3)	O20—C21	1.459 (3)
C7—C12	1.507 (3)	C21—H21A	0.9700
C8—C9	1.518 (3)	C21—H21B	0.9700
C8—C13	1.468 (3)	C21—C22	1.479 (5)
C9—H9	0.9800	C22—H22A	0.9600
C9—C10	1.521 (3)	C22—H22B	0.9600
C10—C11	1.353 (3)	C22—H22C	0.9600
C10—C18	1.477 (3)	C23—H23A	0.9600
C11—C23	1.496 (3)	C23—H23B	0.9600
C12—H12A	0.9600	C23—H23C	0.9600
C12—H12B	0.9600		
			
C5—S1—C2	94.05 (12)	H12B—C12—H12C	109.5
S1—C2—H2	123.5	O14—C13—C8	123.8 (2)
C3—C2—S1	113.10 (19)	O14—C13—O15	121.5 (2)
C3—C2—H2	123.5	O15—C13—C8	114.7 (2)
C2—C3—C4	110.2 (2)	C13—O15—C16	118.0 (2)
C2—C3—C9	124.8 (2)	O15—C16—H16A	110.2
C4—C3—C9	124.9 (2)	O15—C16—H16B	110.2
C3—C4—H4	123.1	O15—C16—C17	107.4 (2)
C3—C4—C5	113.8 (2)	H16A—C16—H16B	108.5
C5—C4—H4	123.1	C17—C16—H16A	110.2
S1—C5—H5	125.6	C17—C16—H16B	110.2
C4—C5—S1	108.88 (18)	C16—C17—H17A	109.5
C4—C5—H5	125.6	C16—C17—H17B	109.5
C7—N6—H6	115 (2)	C16—C17—H17C	109.5
C7—N6—C11	123.8 (2)	H17A—C17—H17B	109.5
C11—N6—H6	120 (2)	H17A—C17—H17C	109.5
N6—C7—C12	112.6 (2)	H17B—C17—H17C	109.5
C8—C7—N6	118.9 (2)	O19—C18—C10	126.2 (2)
C8—C7—C12	128.5 (2)	O19—C18—O20	121.9 (2)
C7—C8—C9	119.7 (2)	O20—C18—C10	111.8 (2)
C7—C8—C13	125.5 (2)	C18—O20—C21	117.0 (2)
C13—C8—C9	114.60 (19)	O20—C21—H21A	109.2
C3—C9—H9	108.4	O20—C21—H21B	109.2
C8—C9—C3	110.47 (18)	O20—C21—C22	112.2 (3)
C8—C9—H9	108.4	H21A—C21—H21B	107.9
C8—C9—C10	110.90 (18)	C22—C21—H21A	109.2
C10—C9—C3	110.21 (18)	C22—C21—H21B	109.2
C10—C9—H9	108.4	C21—C22—H22A	109.5
C11—C10—C9	119.68 (19)	C21—C22—H22B	109.5
C11—C10—C18	120.6 (2)	C21—C22—H22C	109.5
C18—C10—C9	119.6 (2)	H22A—C22—H22B	109.5
N6—C11—C23	113.4 (2)	H22A—C22—H22C	109.5
C10—C11—N6	118.6 (2)	H22B—C22—H22C	109.5
C10—C11—C23	128.0 (2)	C11—C23—H23A	109.5
C7—C12—H12A	109.5	C11—C23—H23B	109.5
C7—C12—H12B	109.5	C11—C23—H23C	109.5
C7—C12—H12C	109.5	H23A—C23—H23B	109.5
H12A—C12—H12B	109.5	H23A—C23—H23C	109.5
H12A—C12—H12C	109.5	H23B—C23—H23C	109.5
			
S1—C2—C3—C4	0.1 (3)	C9—C3—C4—C5	177.0 (2)
S1—C2—C3—C9	−177.09 (17)	C9—C8—C13—O14	−16.0 (4)
C2—S1—C5—C4	−0.1 (2)	C9—C8—C13—O15	161.9 (2)
C2—C3—C4—C5	−0.2 (3)	C9—C10—C11—N6	−9.6 (4)
C2—C3—C9—C8	−23.5 (3)	C9—C10—C11—C23	172.7 (3)
C2—C3—C9—C10	99.4 (3)	C9—C10—C18—O19	−170.8 (3)
C3—C4—C5—S1	0.2 (3)	C9—C10—C18—O20	10.6 (3)
C3—C9—C10—C11	−93.6 (3)	C10—C18—O20—C21	−179.8 (2)
C3—C9—C10—C18	82.7 (2)	C11—N6—C7—C8	15.7 (4)
C4—C3—C9—C8	159.7 (2)	C11—N6—C7—C12	−164.1 (3)
C4—C3—C9—C10	−77.4 (3)	C11—C10—C18—O19	5.5 (4)
C5—S1—C2—C3	0.0 (2)	C11—C10—C18—O20	−173.1 (2)
N6—C7—C8—C9	7.6 (4)	C12—C7—C8—C9	−172.6 (3)
N6—C7—C8—C13	−177.6 (2)	C12—C7—C8—C13	2.2 (4)
C7—N6—C11—C10	−14.7 (4)	C13—C8—C9—C3	−80.8 (2)
C7—N6—C11—C23	163.4 (3)	C13—C8—C9—C10	156.7 (2)
C7—C8—C9—C3	94.5 (3)	C13—O15—C16—C17	177.2 (3)
C7—C8—C9—C10	−28.0 (3)	O14—C13—O15—C16	−1.1 (4)
C7—C8—C13—O14	169.0 (3)	C18—C10—C11—N6	174.1 (2)
C7—C8—C13—O15	−13.1 (4)	C18—C10—C11—C23	−3.6 (4)
C8—C9—C10—C11	29.0 (3)	C18—O20—C21—C22	85.3 (3)
C8—C9—C10—C18	−154.6 (2)	O19—C18—O20—C21	1.5 (4)
C8—C13—O15—C16	−179.1 (2)		

### Hydrogen-bond geometry (Å, º)

*Cg*1 is the centroid of the thiophene S1/C2–C5 ring.

**Table:**

*D*—H···*A*	*D*—H	H···*A*	*D*···*A*	*D*—H···*A*
N6—H6···O14^i^	0.82 (4)	2.19 (4)	3.010 (3)	176 (3)
C5—H5···O19^ii^	0.93	2.52	3.220 (4)	133
C9—H9···O20	0.98	2.36	2.739 (3)	102
C12—H12*B*···O15	0.96	2.33	2.768 (3)	107
C23—H23*C*···O19	0.96	2.42	2.826 (4)	105
C17—H17*C*···*Cg*1^iii^	0.96	2.79	3.720 (4)	162

Symmetry codes: (i) *x*, *y*−1, *z*; (ii) −*x*, *y*+1/2, −*z*+1/2; (iii) −*x*+1, −*y*+2, −*z*+1.
